# Tissue Doppler echocardiography – A case of right tool, wrong use

**DOI:** 10.1186/1476-7120-2-12

**Published:** 2004-08-12

**Authors:** George Thomas

**Affiliations:** 1 Department of Cardiology, Indira Gandhi Co-operative Hospital and the Co-operative Medical College, Kochi 682 020, India

**Keywords:** Echocardiography, Doppler studies, Tissue Doppler, Flow Doppler, uses, misuse

## Abstract

**Background:**

The developments in echocardiography or ultrasound cardiography (UCG) have improved our clinical capabilities. However, advanced hardware and software capabilities have resulted in UCG facilities of dubious clinical benefits. Is tissue Doppler echocardiography (TDE) is one such example?

**Presentation of the hypothesis:**

TDE has been touted as advancement in the field of echocardiography. The striking play of colors, impressive waveforms and the seemingly accurate velocity values could be deceptive. TDE is a clear case of inappropriate use of technology.

**Testing the hypothesis:**

To understand this, a comparison between flow Doppler and tissue Doppler is made. To make clinically meaningful velocity measurements with Doppler, we need prior knowledge of the line of motion. This is possible in blood flow but impossible in the complex myocardial motion. The qualitative comparison makes it evident that Doppler is best suited for flow studies.

**Implications of the hypothesis:**

As of now TDE is going backwards using an indirect method when direct methods are better. The work on TDE at present is only debatable 'research and publication' material and do not translate into tangible clinical benefits. There are several advances like curved M-mode, strain rate imaging and tissue tracking in TDE. However these have been disappointing. This is due to the basic flaw in the application of the principles of Doppler. Doppler is best suited for flow studies and applying it to tissue motion is illogical. All data obtained by TDE is scientifically incorrect. This makes all the published papers on the subject flawed. Making diagnostic decisions based on this faulty application of technology would be unacceptable to the scientific cardiologist.

## Background

Echocardiography or Ultrasound Cardiography (UCG) is a key investigation in cardiology. Its non-invasive nature makes it a widely acceptable and safe form of investigation. In many cases it has surpassed cardiac catheterisation in diagnostic yield and has become the investigation of choice.

The rapid development of UCG has made available to the clinician newer insights into the anatomy and physiology of the heart. With the availability of digital technology it is possible to manipulate raw data in different ways. This has also spawned UCG facilities of dubious clinical benefits. Tissue doppler echocardiography (TDE) is one such example.

When we consider UCG we are interested in 2 distinct aspects of the heart:

1. Structure 2. Blood flow. Both these ultimately indicate cardiac function. B-mode/M-mode imaging or simply Ultrasound Imaging (USI) studies structure. Blood flow is studied by Doppler studies (DS). In each stream every development should incrementally improve our understanding of the heart, its structure and function. Or the development should improve the ease of operation or permit better documentation.

In USI we had the following developments and each development added to the diagnostic yield. M-mode – temporal resolution; 2D – spatial resolution; Harmonic imaging – improvement in image quality; B-color – enhanced tissue perception; Ultrasonic tissue characterization and acoustic quantification – quantitative analysis of tissue; Omni-plane M-mode – temporal resolution at different segments and 3D imaging – another dimension of imaging.

In DS too we had many developments, each improving our perception. Continuous wave (CW) – detected and measured high velocity flows; Pulse wave (PW) – located the abnormal flow; Color flow mapping (CFM) – made it possible to 'image' blood flows and power amplitude Doppler – to study vascular flow. Contrast, trans-oesophageal and intravascular UCG are the clinical extensions and exploitation of these developments

What we see in TDE is a retrograde development. In the context of UCG, DS is an indirect method to study blood flow since USI in its present state cannot image fast moving blood cells. Spontaneous echo contrasts could mark the beginning of USI of blood cells. By using TDE we are using an indirect method where direct USI methods are better. Besides, TDE is an inappropriate application of the Doppler methodology.

## Presentation of the hypothesis

TDE is a by-product of color flow mapping (CFM) technology. In CFM, tissue signals are suppressed and flow signals are analyzed. The reverse is true in TDE. Doppler velocities associated with tissue motion are slower than blood flow. Flow signals are eliminated on the basis of signal amplitude. The amplitude of tissue motion is about 40 db greater than the corresponding flow amplitude. Blood flow imaging applies a high pass filter to exclude the strong but low frequency tissue signals and other 'noise' before the signal is input into an autocorrelator that estimates velocity.[1] Erroneous filter settings could cause the autocorrelator to include components of tissue signals so that tissue velocities become encoded. This principle has been legitimized to color code tissue motion and we get an 'image' which is entirely Doppler information. In this discussion TDE includes both pulse tissue Doppler and color tissue Doppler (also known as Doppler tissue imaging or tissue velocity imaging).

The first report on the use of TDE appeared in 1989.[2] However the real development and the widespread use of this technique started after the publication of the validation work of TDE setting the 'scientific' basis for the quantitative analysis of myocardial velocities in real time.[3] There are several papers on the use of TDE.[4,5] Various values and indices are already in place.[6,7] Over the past few years technological advances in TDE like curved M-mode, strain rate imaging and tissue tracking have been developed. These are in addition to modalities like the phase imaging, amplitude imaging and acceleration imaging.[8] Text books on TDE has also been added to the number of books already available on UCG.[9] Even now there are a number of papers on tissue Doppler echocardiography (TDE) appearing in leading journals.[10] Is TDE methodology correct? Is it in agreement with the Doppler principles? Has TDE made any tangible improvement in the already available UCG techniques? Has TDE improved our diagnostic yield? This article presents the hypothesis that TDE is a flawed application of Doppler and hence data collection with TDE is erroneous.

## Testing the hypothesis

The article explains the flaws in tissue Doppler echocardiography. As the concept itself is flawed, *all data using this modality is flawed*. You cannot even think of designing a study to prove the point because the mensuration technique itself is wrong. So we have to use the scientific methods of comparison and *reductio ad absurdum *to verify it. Any new modality of diagnosis or treatment should be compared with the existing systems. In clinical trials we usually employ *quantitative *comparison with statistical methods. In this case, since the data acquisition is flawed we have to use a *qualitative *comparison. This is accepted practice in clinical medicine – It is like comparing a weak limb with the strong one in neurology. One also needs to understand the Doppler methodology to test the hypothesis.

### The Doppler methodology

To estimate clinically significant peak Doppler velocity the following steps are required:

1. You must have *prior knowledge *of the line of motion of the object. Only if you know this line of motion, you can apply the interrogating signal along the path of motion. This is because of the directional bias of Doppler.

2. Next you will know the direction of motion – whether the object is moving towards the interrogating signal or away.

3. Only after the first two steps you can measure the clinically significant peak velocity.

Based on the above discussion it is clear that the following information can be derived from Doppler:

1. Is there motion? Does the object move? This is a random application of Doppler. This is one aspect of tissue Doppler. But this information is redundant as we can already see the movement better by ultrasound imaging.

2. What is the direction of motion? Is it moving towards or away from the interrogating signal? This is also redundant information in tissue Doppler for the reason given above.

3. To measure the peak *significant *velocity we must know the clear-cut line of motion. Application of Doppler along this line enables us to measure the meaningful peak velocity.

Thus the primary step is to know the line of motion. This is possible in flow Doppler but impossible in tissue Doppler. For example we know for sure that the blood moves from left atrium to left ventricle through the mitral valve. At the mitral valve this is a unique free linear motion towards the apex. This produces a clinically significant velocity. That is why we place the sample volume at the mitral valve from apical views so as to align the beam in the line of *anticipated *flow. That is also why we *do not *measure the mitral flow velocity from the parasternal views. Next we come to learn whether the blood is flowing towards or away from the transducer. After all these steps it is possible to measure velocity. In tissue Doppler we can measure several velocities in several directions. But we can never know which is the clinically significant peak velocity.

### Flaws in TDE

TDE is a clear case of misuse of technology. To understand this, a comparison between flow Doppler and tissue Doppler is in order. CFM allows us to 'see' what we cannot see with ultrasonic 'eyes' hence its value is great. In color TDE we see more or less what we already see by USI hence its value is marginal. In CFM the anatomic landmarks are intact as the color is superimposed on the B-mode image. In color TDE the B-mode is eliminated and the entire 'picture' is Doppler information. It is difficult to determine the different anatomic regions on TDE. For example it would be very difficult to delineate the blood-endocardial boundary. In fact, the word 'tissue Doppler' is a misnomer and this is one of the reasons for the prevailing confusion. It gives the impression that only myocardial tissues are studied. The appropriate term would have been low velocity Doppler. Any movement in the low velocity range will be detected by 'tissue Doppler'. Myocardial tissue movement is just one among them. The discriminatory power of this modality is unsatisfactory.

Myocardial motion is very complex and not amenable to Doppler studies. In the cardiac motion there are translational, rotational and deformational movements. Besides many tissues near the heart move – due to transmitted cardiac motion, vessel pulsation, respiratory motion and involuntary muscle movements and these interact with cardiac motion further and cause false Doppler shifts.[1] Velocity is a vector quantity and so Doppler interrogation at one point will determine the velocity of the resultant of all these movements projected in the line of the Doppler beam with angle correction. Similarly at a particular point there are movements in several axes and we can never predict the sum resultant vector. Even if known, the resultant is accurately recorded only if it is in the line of the Doppler beam. This is due to the inherent problem of directional bias. It is like measuring the length of a twisted rod directly with a straight rigid ruler. In the case of strain rate imaging, the protagonists have substituted a vector quantity (velocity) for a scalar quantity (length) in the original formula of strain calculation.[11] This would be mathematically unacceptable. Cardiac motion becomes more complicated in the presence of wall motion abnormalities.

Blood flow is simple and suitable for Doppler study. In flow Doppler at a particular point the linear projectile motion of free moving blood cells are studied. At the current interrogation points there is a unique unidirectional flow in one part of the cardiac cycle. For example in mitral valve Doppler interrogation, the unique directional signal is obtained only in diastole. If there is a signal in the in systole, it becomes abnormal and this information has great value. In TDE the to- and- fro motion (systole and diastole) of a tethered interconnected syncytium of myocytes is imaged. Such information is useless. This can be even otherwise seen and analyzed by USI.

In flow Doppler there are definite 'points of interrogation', which are the normal and abnormal orifices. In TDE there are no such definite points. While in flow Doppler higher velocities are studied, TDE studies lower velocities i.e. the study of hypo functioning myocardial segments. Lower velocities are difficult to appreciate. The higher velocities are easier to appreciate due to aliasing and variance. There are no such indicators for low velocity. Thus hypo function is difficult to analyze. The derivations from flow Doppler are based on accepted hydrodynamic formulae (like Bernoulli's equation) and allows us to get orifice size, amount of flow and pressure gradients, which are clinically of great importance. The derivations from TDE are useless. Thus *Doppler in its present form, is best suited to study flow.*

## Implications of the hypothesis

TDE has shown some promise in the Wolff-Parkinson-White syndrome.[12] But even here by having a high frame rate or by using the omni plane M-mode, we could be able to locate the pre-excited tissue by USI. Diastolic dysfunction in pseudo-normalized mitral Doppler spectrum and in atrial fibrillation are other areas where TDE is found useful.[13,14] Here the benefits are marginal as there are other parameters to study diastolic dysfunction.[15] Besides, the determination of diastolic dysfunction by Doppler in clinical practice may not be sacrosanct.[16] It has been used as a method to differentiate between constrictive pericarditis and restrictive cardiomyopathy.[17] Here its role is supplementary and does not provide critical distinction. The main role of TDE as a method of detecting regional wall motion abnormalities has been stressed.[18] Here again it is complementary to routine USI. TDE is being used in the study of cardiac transplant rejection.[19] In this situation the results are not clear and there are other better UCG methods to study rejection.[20,21] TDE has been advocated in planning and follow-up of cardiac re-synchronization therapy.[22,23] Here again the conventional methods are adequate[24,25] and the false-positive data (see below) may be confounding. Besides several issues regarding the therapy need to be settled.[26] Mitral annular tissue velocities have been used in the determination of left ventricular filling pressures.[27] Here again an 'hydrodynamic' information (fluid pressure) is derived from non-hydrodynamic (tissue velocity) measurement. Mitral annulus is a circular area. As mentioned earlier here also we cannot define the precise point of interrogation. An infinite number of values can be obtained all around the mitral annulus. *It is important to note that in all the above papers the data acquired is flawed due to the basic problem in technology application*. *Hence the correlations obtained in these papers are spurious or non-sense correlations*. One paper presents a misuse of statistics when tissue Doppler velocities have been correlated with myocardial interstitial fibrosis and myocyte interstitial beta-adrenergic receptor density.[28] By analogy, using a little statistical jugglery, we could find correlation between blood cholesterol levels and flow Doppler indices. All these papers are mentioned just for completeness. Accepting the data in these papers would be tantamount to rejecting the Doppler principle and methodology. As of now TDE is going backwards using an indirect method where direct methods are available. The work on TDE at present is only debatable 'research and publication' material and does not translate into tangible clinical benefits.

The prime use of TDE is to study regional wall motion abnormalities. It is claimed that it is possible to quantify wall motion at rest and during stress. However with the above-mentioned limitations it is unlikely to prove more effective than USI. The measurements are ultra sensitive and represent gross movements rather than myocyte contractions. With the advent of omni-plane M-mode it is possible to get temporal resolution in inaccessible planes and study wall motion abnormalities in a better way.

The Doppler methods use mathematical formulae to indirectly study motion that cannot be directly measured. Examples are movements of stellar bodies and in the present context movements of blood cells. We use the indirect method based on Doppler principle because we cannot 'image' blood cells by USI. When the imaging of blood cells become possible, Doppler studies would probably be relegated to the background. Color TDE would have been valuable if direct USI was not available.

Tissue Doppler has been a disappointing modality in clinical ultrasound cardiology. This is due to the basic flaw in the application of the principles of Doppler. Doppler is best suited for flow studies and applying it to tissue motion is not acceptable. In flow Doppler we suppress the tissue 'noise' and display flow.[1] In TDE it is the other way round. Besides TDE is ultra sensitive and so the information gathered is almost useless (too many false positive information). In fact excellent cardiac waveforms can be obtained by placing the sample volume just outside the cardiac region! This is also the reason why it is useless for even studying the temporal aspects of the cardiac cycle and waveform analysis. Once the foundation of a modality is wrong, all derivations tend to be wrong.

However the author is of the view that TDE could probably have some role in diagnostic UCG. What is required is that Doppler should be used for the purpose it was intended i.e. to study blood flow. Thus we will have 2 types of Doppler studies: High velocity flow Doppler (HVFD) studies and Low velocity flow Doppler (LVFD) studies. Conventional Doppler would be HVFD. TDE would be LVFD and we would be imaging and analyzing very low velocity flows. The possible uses of color LVFD studies would be the study of low flow thrombogenic states, flow in the atria, the blood-endocardial/endothelial interface, low velocity turbulences and blood flow in organs. Pulse wave spectral LVFD could be useful for the detection of low velocity turbulences as a cause for murmurs (not detectable with HVFD). These are *potential *areas for investigation and research.

It is time to look at TDE on more realistic terms. As a new modality of imaging it appears exciting. But its real clinical utility is doubtful. TDE does not give any additional information over the conventional modalities. In fact due to the above-mentioned deficiencies it could give misleading information. Making diagnostic and therapeutic decisions based on this faulty application of technology would be unacceptable to the scientific cardiologist.

## List of abbreviations used

CFM Color Flow Mapping

CW Continuous Wave

DS Doppler Studies

HVFD High Velocity Flow Doppler

LVFD Low Velocity Flow Doppler

PW Pulse Wave

TDE Tissue Doppler Echocardiography

UCG Ultrasound Cardiography

USI Ultrasound Imaging

## Competing interests

None declared.
